# *In vitro* and *in vivo* analysis of the biocompatibility of two novel and injectable calcium phosphate cements

**DOI:** 10.1093/rb/rby027

**Published:** 2018-12-19

**Authors:** Dan Meng, Limin Dong, Yafei Yuan, Qingsong Jiang

**Affiliations:** 1 Department of Prosthodontics, Beijing Stomatological Hospital, School of Stomatology, Capital Medical University, Beijing, China; 2 Beijing Key Laboratory of Fine Ceramics, Institute of Nuclear and New Energy Technology, Tsinghua University, Energy Science Building, Beijing, China

**Keywords:** calcium phosphate cements, biocompatibility, injectable scaffold, cellular effects

## Abstract

Calcium phosphate cements (CPCs) have been widely used as bone graft substitutes for many years. The aim of this study was to evaluate the biocompatibility of two novel injectable, bioactive cements: β-tricalcium phosphate (β-TCP)/CPC and chitosan microsphere/CPC *in vitro* and *in vivo.* This was accomplished by culturing mouse pre-osteoblastic cells (MC3T3-E1) on discs and pastes of CPCs. Cell growth, adhesion, proliferation and differentiation were assessed by 3-(4, 5-dimethylthiazol-2-yl)-2, 5-diphenyltetrazolium bromide and alkaline phosphatase assays as well as by scanning electron microscopy and fluorescence. The effect of CPC paste curing was also evaluated. Implantation of two materials into the muscle tissue of rabbits was also studied and evaluated by histological analysis. Cell analysis indicated good biocompatibility *in vitro.* The fluorescence assay suggested that the cured material discs had no obvious effect on cell growth, while the curing process did. Histological examination showed no inflammatory cell infiltration into soft tissue. These data suggest that β-TCP/CPC and chitosan microsphere/CPC composites may be promising injectable material for bone tissue engineering.

## Introduction

Calcium phosphate cements (CPCs) have been widely studied and applied clinically as bone graft substitutes for dental, craniofacial and orthopedic applications [1–3]. CPCs are self-setting, have moderate compressive strength even at loading sites, are highly biocompatible, and have osteo-conductive properties. These properties are conducive to growth and differentiation of osteoblasts and osteo-progenitor cells [4]. For the dental and craniofacial fields, CPCs are used for mandibular and maxillary ridge augmentation, major reconstructions of the maxilla or mandible after trauma or tumor resection and support of dental implants [5–7]. Unfortunately, CPCs do have disadvantages including poor absorption and low strength, which undermine the clinical therapeutic efficacy of CPCs.

β- Tricalcium phosphate (TCP)/CPC and chitosan microsphere/CPC are two novel self-setting bioactive cements produced by Institute of Nuclear and New Energy Technology, Tsinghua University (Beijing, China). β-TCP or chitosan microspheres increase CPC cell compatibility *in vitro* and bio-functionalization *in vivo.* Our previous studies demonstrated improvements in degradation and osteo-conductive properties *in vivo* by porous chitosan microsphere/CPC [8].

An appropriate bone tissue engineering scaffold is important for attachment, proliferation and differentiation of osteoblasts [9, 10]. In this study, *in vitro* experiments were performed to assess adhesion, proliferation and differentiation of osteoblasts as well as effects on cells during the curing process of β-TCP/CPC and chitosan microsphere/CPC. Implantation of two materials into muscle tissue of rabbits was also studied to evaluate *in vivo* biocompatibility. The purpose of this investigation was to provide a theoretical basis for future studies.

##  Materials and methods

###  Preparation of CPC materials

In our study, the control α-TCP/CPC powder was composed of α-TCP [α-Ca_3_(PO_4_)_2_], calcium dihydrogen phosphate monohydrate [Ca(H_2_PO_4_)_2_H_2_O] and calcium carbonate (CaCO_3_) in a 10: 3.5: 1.5 molar ratio. These powders were added with β-TCP (50%, w/w) to form a β-TCP/CPC powder. A sodium dihydrogen phosphate (NaH_2_PO_4_)/sodium hydrogen phosphate (Na_2_HPO_4_) solution in equal molar ratio was the liquid phase. The chitosan microsphere/CPC powders were the α-TCP/CPC powders added with chitosan microspheres (10%, w/w). The chitosan (DD > 90%, MW = 57 000) microspheres were prepared by a liquid-phase suspension method, were 100–400 μm in diameter [11, 12]. The chitosan microsphere/CPC liquid was composed of the above liquid to which a sodium bicarbonate (NaHCO_3_) solution and a sodium alginate [(C_6_H_7_O_6_Na)n] solution (1.5%, w/v) were added. The solid–liquid ratio was all 1 g/ml. The microstructure of the scaffolds was observed by scanning electron microscopy (SEM) (Quanta 200 FEG, FEI, Netherlands). All materials were provided by Tsinghua University.

The above three powders were mixed with corresponding liquids at a powder to liquid ratio of 1: 1. They were poured into a cylindrical mold (Ø10  mm, height 5 mm) to fabricate setting discs for SEM, 3-(4, 5-dimethylthiazol-2-yl)-2, 5-diphenyltetrazolium bromide (MTT) and alkaline phosphatase (ALP) assays. They were poured into a cylindrical mold (Ø 10 mm, height 1 mm) for fluorescence and a cylindrical mold (Ø 4 mm, height 5 mm) for animal implantation. These setting discs and powders and liquids of CPCs were sterilized by ^60^Co γ-radiation with 25 kGy and stored at 4°C.

###  Cell culture

MC3T3-E1 cells (Cell Resource Center, IBMS, CAMS/PUMC, Beijing, China) were cultured in advanced minimal essential media containing 10% fetal bovine serum ( Gibco, Auckland, New Zealand) and 1% penicillin-streptomycin in an incubator at 37°C and 5% CO_2_. Osteogenic medium consisted of the above medium plus 10 nM dexamethasone, 10 mM β-glycerophosphate, and 50 μM ascorbic acid (Sigma, Beijing, China). The cells of the fourth and fifth passage that grew in a log growth phase were applied to the study. At 90% confluence, cells were harvested, centrifuged (120 g, 3 min) and cell suspensions prepared. The cells were then placed in new culture dishes at a density of 2–5 × 10^4^ cells/ml.

###  Observation of cellular morphology

Before seeding cells, three discoid materials (*n* = 4 for each group) were placed into wells of a 96-well plate. Cells were then seeded on the discs samples at a density of 2 × 10^4^/well and cultured in a 5% CO_2_ at 37°C and observed with an inverted microscope (CKX53, Olympus, Tokyo, Japan). After 7 days, the samples were washed with a phosphate buffered saline (PBS) buffer solution, fixed with a 3% glutaraldehyde solution, and then dehydrated in an ascending series of ethanol and dried in the air. Each sample was observed by SEM and digitally photographed.

###  Cell proliferation analysis

Proliferation of cells treated with α-TCP/CPC, β-TCP/CPC, chitosan microsphere/CPC and a blank control were evaluated by MTT analysis at Days 1, 4, 7 and 10 (*n* = 4 for each group). The MTT (Amresco, USA) solution was added and then incubated for 4 h. The media was replaced with dimethyl sulfoxide ( USA) and the absorbance measured at 570 nm with an ELX Ultra Microplate Reader (Bio-tek, USA). Data were averaged.

###  Cell differentiation analysis

Differentiation of cells treated with α-TCP/CPC, β-TCP/CPC, chitosan microsphere/CPC and a blank control were evaluated for ALP activity at Days 7 and 14 (*n* = 4 for each group). ALP activity was detected using a specific assay kit (Nanjing Jiancheng, China). Light absorption of the solution at 405 nm was measured and data averaged.

###  Fluorescence assay

The effect on cells of β-TCP/CPC discs, chitosan microsphere/CPC discs, β-TCP/CPC pastes, chitosan microsphere/CPC pastes and a blank control was evaluated by fluorescence (*n* = 4 for each group). Disc samples were placed into wells of a 24-well plate. Cells were then seeded on the discs at a density of 2 × 10^4^/well and cultured in a 5% CO_2_ incubator at 37°C for 24 h. Then the discs were washed with a PBS buffer solution, fixed and air dried. Discs samples were stained with 0.01% euchrysine. The β-TCP/CPC pastes and chitosan microsphere/CPC pastes were fabricated and spread to a height of 1 mm in 24-well plates. Cells were then seeded onto the pastes at a density of 2 × 10^4^/well, cultured for 24 h, and stained with 0.01% euchrysine. All samples were observed with a fluorescence microscope (BX53, Olympus, Tokyo, Japan) and photographed digitally.

###  Implantation of material into the muscle tissue of rabbits

All surgeries were performed by a protocol approved by the Animal Welfare Committee of Capital Medical University. Six New Zealand White rabbits (male, 4-weeks -old, with an average weight of 2.6 kg) were used in our study.

The rabbits were placed in an abdominal position and back muscles exposed after anesthesia. Samples of β-TCP/CPC and chitosan microsphere/CPC, 4 mm in diameter and 5 mm in height, were implanted into the back muscles on either side. The wounds were sutured and penicillin (240 000 IU) was used for 3 days. The procedure was performed in a sterile operating room. Animals were sacrificed by air embolism 4 and 8 weeks after surgery (*n* = 3 per group) and tissue samples from the back muscles harvested. The samples were fixed, decalcified, washed, dehydrated and then embedded in paraffin. Sections (5 μm) of each of the samples were stained with hematoxylin and eosin (H&E) and were observed with a microscope (BM1000, Nikon, Japan) and photographed digitally.

###  Statistical analysis

SPSS 20.0 software (SPSS Inc., Chicago, IL, USA) was used for data input and analysis. MTT and ALP assays were assessed by double-factor variance analysis and independent t-test. The fluorescence assay was assessed by double-factor variance analysis. A *P* < 0.05 was considered statistically significant.

##  Results

###  Observation of scaffold microstructure

**Figure 1:**
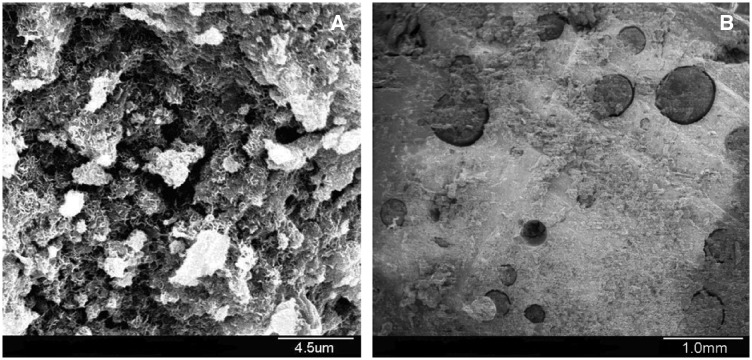
SEM micrographs of the surface morphology of β-TCP/CPC **(A)** and chitosan microsphere/CPC scaffolds **(B)**

SEM micrographs of cross-sectioned β-TCP/CPC and chitosan microsphere/CPC specimens are presented in Fig. 1. Nano-scale microcrystals grew on β-TCP/CPC surfaces uniformly (Fig. 1A). The size of the crystal was generally 300–500 nm, and the interlacing connections between the crystals formed a network, increasing strength. The chitosan microspheres and CPC combined with each other, with small gaps around the microspheres (Fig. 1B).

###  Observation of cellular morphology

**Figure 2:**
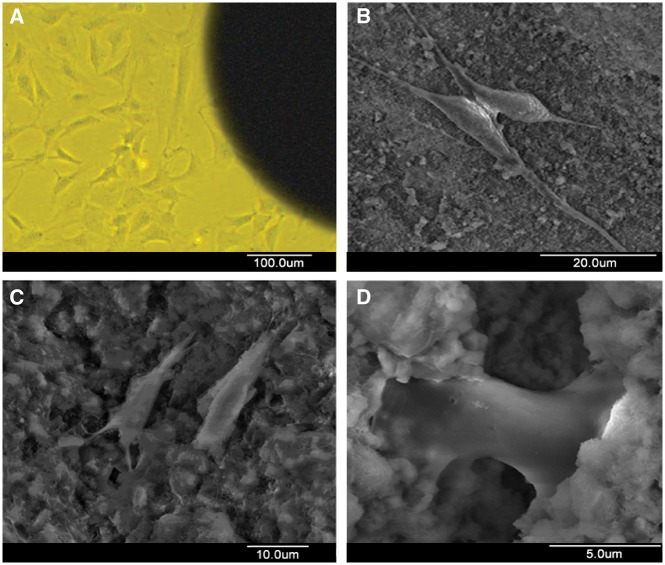
Inverted microscope and SEM images of cells on three types of material: **(A, B)** β-TCP/CPC, **(C)** α-TCP/CPC, **(D)** chitosan microsphere/CPC

Cells attached firmly to the three types of material. Cells had spindle-shaped morphologies, full and smooth, with many filopodia. There were cells adherent to the surface of the materials as well as to the inside of the materials (Fig. 2). There were reticular fibroblast-like substances on the surface of the cells, as judged by high power microscopic observation. Collagen may have been secreted by osteoblasts.

###  Cell proliferation analysis

**Figure 3:**
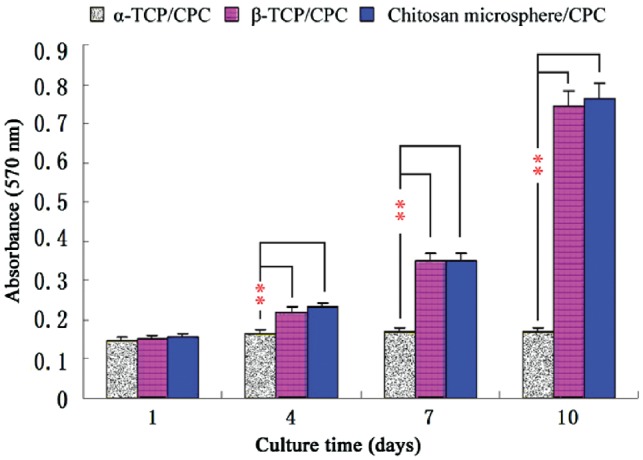
MTT Absorbance values of three types of material on different days (** *P* < 0.01)

As shown in Fig. 3, the MTT assay showed no significant differences among the three groups at Day 1. MTT values significantly increased with β-TCP/CPC and α-TCP/CPC or chitosan microsphere/CPC and α-TCP/CPC at Days 4, 7 and 10 (*P* < 0.01). However, there was no significant difference between the β-TCP/CPC and the chitosan microsphere/CPC group.

###  Cell differentiation analysis

**Figure 4:**
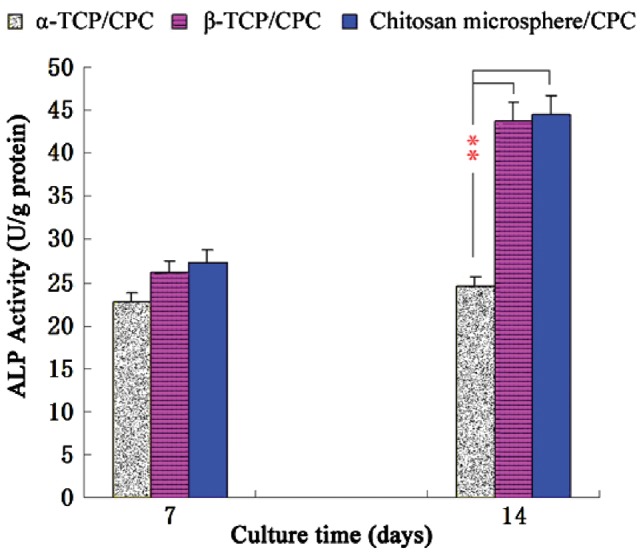
ALP Activity values of three types of material on different days (**P < 0.01)

As shown in Fig. 4, ALP quantitative assay data showed that ALP activity in the β-TCP/CPC and chitosan microsphere/CPC group was higher than that of the α-TCP/CPC group at Day 7. However, there was no significant difference among the three groups. ALP activity significantly increased with β-TCP/CPC and α-TCP/CPC or chitosan microsphere/CPC and α-TCP/CPC at Day 14 (*P* < 0.01).

###  Fluorescence assay

**Figure 5:**
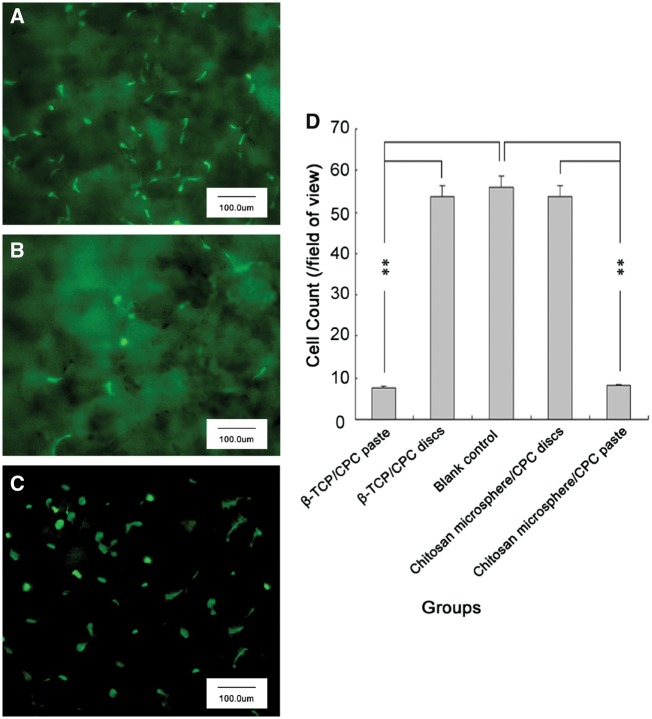
Fluorescence images of chitosan microsphere/CPC discs **(A)**, chitosan microsphere/CPC paste **(B)**, and the blank control **(C)**, as well as cell counts the different groups **(D)** (**P < 0.01)

As shown in Fig. 5, stronger fluorescence was observed in the discs and the blank control, with weaker fluorescence for the CPC pastes. The cell counts for β-TCP/CPC discs, chitosan microsphere/CPC discs and the blank control group were significantly higher than the β-TCP/CPC paste and chitosan microsphere/CPC paste group (*P* < 0.01). There was no significant difference among groups of β-TCP/CPC discs, chitosan microsphere/CPC discs, or blank controls. These data suggest that the cured material discs had no obvious effect on cell growth, while the curing process did.

###  Histological examination

**Figure 6:**
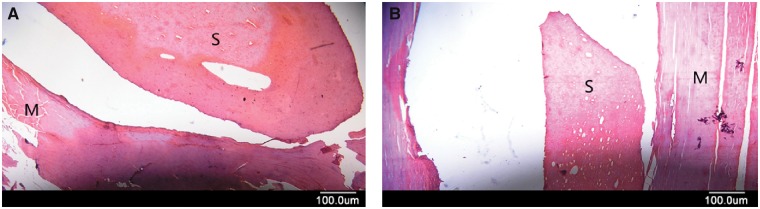
H&E staining of the implantation areas of β-TCP/CPC. **(A)** 4-week group. **(B)** 8-week group. M, muscle; S, scaffold material

The materials in the muscles tissues were evaluated histologically. Representative histological images of β-TCP/CPC are shown in Fig. 6 (H&E staining). There was no significant difference between the 4-week group (Fig. 6A) and the 8-week group (Fig. 6B). The muscle tissue around the implantation sites was normal. The implantations were surrounded by a thin, incomplete layer of connective tissue that did not adhere tightly to the surrounding muscle tissue. In tissue sections, partial fibrous membranes were separated from muscle tissue. The inner surface of connective tissue was smooth, without abnormal protuberance and the fiber bundles were arranged in parallel and orderly. Muscle tissue could be observed in transverse muscle lines, with no structural disorder. No osteoblasts were found in the connective tissue and no similar bone structure was observed. No growth of connective tissue and blood vessels to implantation materials was observed. No inflammatory cell infiltration was found in soft tissue and no tissue degeneration was found.

##  Discussion

β-TCP/CPC and chitosan microsphere/CPC composites are two novel, injectable bone cement materials. The study of biocompatibility has always been the focus of tissue engineering research [13, 14]. He *et al. *[15] studied surface treatment techniques, such as plasma treatment, that immobilized bioactive molecules on CPC/polymer scaffolds to increase wettability, roughness and porosity in order to boost a cellular response. Vater *et al.* [16] investigated growth and osteogenic differentiation of human bone marrow stromal cells (hBMSCs) on Biocement D/collagen composites, which had been modified with osteocalcin and O-phospho-L-serine. Vater *et al.* demonstrated that initial hBMSC adhesion and proliferation is induced by osteocalcin. Perez *et al. *[17] studied a bone inspired material produced by incorporating collagen in the liquid phase into a α-TCP cement, either in solubilized or in fibrilized form. Cell adhesion and proliferation were increased by the composites.

The success of cell seeding depends on cell seeding technique, scaffold structure and relates to the capability of scaffold material to favor cell attachment and proliferation [18]. In this study, MTT values and ALP activity for β-TCP/CPC and chitosan microsphere/CPC were higher than that of α-TCP/CPC. This closely related to the surface properties of these two materials. On the surface of β-TCP/CPC and chitosan microsphere/CPC, there is a layer of carbonated hydroxyapatite (HA) that consists of tiny grains and defective structures—that are bone-like apatite [19]. The similar composition and structure of the bone mineral phase and bone-like apatite allow osteoblasts to preferentially proliferate on the composite surface. Bone-like apatite improves the surface activity of the material and facilitates cell adhesion and proliferation [20, 21].

In addition to the surface properties of the material, a large number of penetrating micropores for β-TCP/CPC and chitosan microsphere/CPC also facilitate the interaction of cells and materials. In several previous studies, pore size was shown to have an important influence on the growth of cells [22]. Micropores increase the contact area between material and cells, providing sufficient space for the growth of cells [23]. In α-TCP/CPC, there are very few pores >10 μm. Such pores are not conducive to the attachment of pseudopodia. Due to larger average pore size, the specific surface area of β-TCP/CPC and chitosan microsphere/CPC is greater than that of α-TCP/CPC, especially with the addition of chitosan microspheres. When specific CPC surface area and surface roughness are increased, more space is provided for the extracellular matrix, and this rough surface improves cell adhesion.

Studies have found that suitable Ca^2+^ and Mg^2+^ ions can promote cell attachment, growth and differentiation due to cell adhesion molecules on cell surfaces [24]. These ions are produced by cells, are involved in signal transduction and cellular activation, as well as the expansion and movement of cells, the growth and differentiation of cells and other important physiological processes [25]. The role of most cell adhesion molecules depends on divalent ions such as Ca^2+^ and Mg^2+^. β-TCP particles are present in β-TCP/CPC. In the cell culture media, these β-TCP particles will slightly dissolve, increasing the concentration of Ca^2+^ ions near the surface of the material. These Ca^2+^ ions promote the function of cell adhesion molecules. Adhesion molecules make it easier for cells to adhere, grow and differentiate. Thus, cells show better cell affinity for β-TCP/CPC than α-TCP/CPC.

Recently, CPC cellular effects have been shown to be due to fluctuations in pH and ion concentration during the curing process [26]. Simon *et al. *[27] studied the effects of CPC pastes on MC3T3-E1 cells. They found that when CPC pastes were in direct contact with cells, there was a significant effect on cell number. However, if the material was separated from the cells with a cover glass, CPC pastes did not have an effect on the MC3T3-E1 cells. In this study, when cells were cultured on the surface of two CPC pastes for 24 h, the number of cells was significantly reduced compared with corresponding CPC discs and a blank control, confirming that CPC pastes influence cells during the curing process. However, our animal implantations experiments showed that CPC into the back muscles of rabbits after 4 and 8 weeks resulted in no inflammatory cell infiltration around the material, nor were osteogenic phenomena observed. Those studies showed that transient changes in the microenvironment, caused by the CPC curing process, may be a major factor that adversely affects cells, especially when CPCs particles are within close contact. When two kinds of CPCs were mixed in either solid or liquid form, curing was rapidly exothermic. The rate of heat release peaked at ∼5 min after the curing reaction began. Heat release gradually decreased until about 50 min. After the curing reaction begins, calcium phosphate salt first dissolves. At this time, the concentration of Ca^2+^ and PO_4_^3^^−^ ions in the paste rapidly increases [28]. The dissolution of β-TCP also releases Ca^2+^, PO_4_^3^^−^ and HPO_4_^3^^−^ ions. After curing for 1 h, a large quantity of HA microcrystals precipitate, although some unreacted raw material particles do remain. The solidification process is a dissolution–precipitation crystallization process [29]. Water is required for the process. The reaction process also releases H^+^, so the pH and ion concentration of the surrounding environment change during the curing reaction [30]. In conclusion, changes in the pH and ion concentration around the cells as well as changes in temperature during curing may be the main factors affecting cells.

Implanting cells and bone cement materials into the body is an exciting area of tissue engineering research. Development of a method for protection of implanted cells, when injected with CPC materials, is an important direction for future research.

##  Conclusion

Two novel, absorbable bioactive materials of β-TCP/CPC and chitosan microsphere/CPC were shown to promote adhesion, proliferation and differentiation of osteoblasts and had good biocompatibility in muscles of animals. These effects were related to surface properties and structures of the biomaterials and have great potential for clinical application. However, the CPC paste curing process has adverse effects on cells, and as a consequence cells must be protected during the early stages of CPC curing.
